# P19 A case of pyrexia of unknown origin - wrong way round!

**DOI:** 10.1093/rap/rkac067.019

**Published:** 2022-09-28

**Authors:** Sandeep Dahiya, Vera Saulite, Frieder Kleemann, Sateesh Nagumantry, Simon Bulley

**Affiliations:** North West Anglia NHS Foundation Trust, Peterborough, United Kingdom; North West Anglia NHS Foundation Trust, Peterborough, United Kingdom; North West Anglia NHS Foundation Trust, Peterborough, United Kingdom; North West Anglia NHS Foundation Trust, Peterborough, United Kingdom; Leeds Teaching Hospitals NHS Trust, Leeds, United Kingdom

## Abstract

**Introduction/Background:**

We present a case of a 43-year-old female with a long-standing psoriatic arthritis (PsA) and background diagnosis of undifferentiated connective tissues disease (uCTD), treated with methotrexate and ixekizumab. She developed systemic symptoms of fever, photosensitive rash, recurrent renal impairment, and was found to have pancytopenia with rapidly rising ferritin. During the course of her disease, her dsDNA antibodies became positive by Crithidia. Bone marrow biopsy showed prominent haemophagocytosis. Coupled with raised soluble CD25 receptor and triglycerides levels, this established the diagnosis of macrophage activation syndrome secondary to either uCTD progression or ixekizumab.

**Description/Method:**

A 43-year-old female nurse with PsA and uCTD (strongly positive ANA, anti-Ro and Anti-La), but no clinical features of CTD, has been treated with methotrexate with good response since 2011. In 2019 she was started on ustekinumab for psoriasis with excellent response. In April 2020 her biologic therapy was changed to ixekizumab due to secondary failure.

In July 2020 she presented with a thigh abscess, upper airway swelling and acute kidney injury. Investigations demonstrated leucopenia, CRP 18mmol/l, and ferritin 764mmol/l. She was treated with oral prednisolone, antibiotics, fluids and underwent incision and drainage of thigh abscess. Repeat tests showed lymphopenia, ESR of 98mm/hr and polyclonal gammaglobulinaemia. MRI thigh ruled out osteomyelitis.

In August 2020 she was readmitted with high fever, florid photosensitive rash and productive cough. Thorough infection screen was negative. Antibiotics were not effective. dsDNA antibodies were now positive by Crithidia. Differential was either natural progression of CTD or secondary to ixekizumab. A bone marrow biopsy showed no evidence of malignancy. Symptoms settled with high dose oral steroids.

She was readmitted two weeks later with recurrence of symptoms, renal impairment, pancytopenia, ferritin >8000mmol/l, raised lactate dehydrogenase and triglycerides. A CT chest, abdomen and pelvis was largely unremarkable. Repeat bone marrow biopsy demonstrated prominent haemophagocytosis. A diagnosis of macrophage activation syndrome (MAS) secondary to CTD was made. Genetic HLH testing (perforin) was normal, soluble CD25R (sCD25R) was markedly raised.

She was treated with IV methylprednisolone, followed by dexamethasone and cyclosporine. Her symptoms settled, cytopenia resolved and ferritin improved. Due to new renal impairment, cyclosporine was discontinued. Renal biopsy demonstrated immunologically mediated glomerulonephritis, likely secondary to CTD. There was mild glomerular damage. Mycophenolate mofetil was added with good response; prednisolone dose was reduced successfully. There was no recurrence of fever or other systemic symptoms, renal function remains stable.

**Discussion/Results:**

MAS is part of a diverse group of inflammatory hyperferritinaemic syndromes. It is considered a secondary form of haemophagocytic lymphohistiocytosis (HLH) developing in patients with autoimmune conditions. Typically MAS is triggered by systemic lupus erythematosus (SLE). To our knowledge, there are no case reports in the literature describing the onset of MAS on the background of PsA. The diagnosis of MAS is invariably challenging due to lack of early biomarkers and non-specific presenting features.

This case illustrates a rare presentation of MAS in a patient with overlapping CTD, psoriasis, and PsA. This patient developed features of lupus as well as a change in her immune profile with positive dsDNA and renal histology in keeping with lupus. This was further complicated by the development of MAS.

Renal function decline was noted prior to starting cyclosporine, and kidney biopsy was not typical for acute interstitial nephritis, making cyclosporine an unlikely culprit. Mild glomerular damage did not warrant immunosuppression beyond steroids.

Fortunately, the patient responded well to IV methylprednisolone and later mycophenolate mofetil. Therefore, anakinra, cyclophosphamide and rituximab were not considered necessary. There is paucity of data on the treatment choices in MAS, with the commonly used HLH 2004 protocol (etoposide, dexamethasone and cyclosporine) showing disappointing results.

With her overlapping immune conditions, the choice of immunosuppressive therapies might also be challenging in future should her symptoms of psoriasis and PsA reoccur after further steroid reduction.

**Key learning points/Conclusion:**

HLS and MAS are poorly understood conditions, thought to be caused by exaggerated inflammatory response to defective granule-mediated cytotoxicity and uncontrolled T-cell activation. This response, in turn, leads to cytokine storm, consequent tissue damage and multi-organ failure.

Hystiocytosis Society has developed diagnostic criteria for HLH, which include a molecular diagnosis consistent with HLH (pathological mutations of PRF1, UNC13D, or STX11), or at least five of eight criteria: fever, splenomegaly, cytopenia, hypertriglyceridaemia/hypofibrinogenaemia, haemophagocytosis on biopsy, low/absent natural killer (NK) cell activity, hyperferritinaemia, elevated sCD25R. Our patient fulfilled six out of eight criteria, making the diagnosis certain.

It should however be noted that these criteria were developed based on paediatric population and their specificity and sensitivity is not tested in adults. Furthermore, haemophagocytosis may not be present in the initial stages of HLH, as in our case, and is not essential for diagnosis. To complicate matters further, haemophagocytosis can be found in bone marrow in other conditions, such as infections and bone marrow disorders.

Extreme hyperferritinaemia is a characteristic feature of HLH, and can be found in only a handful of other conditions, which aids the diagnostic process. These include SLE, hepatocellular injury, adult onset Still’s disease (AOSD), and haematological malignancies. Interestingly, the patient also meets Yamaguchi criteria for diagnosis of AOSD due to having fever, rash, abnormal liver function tests, minor lymphadenopathy, and arthralgia. This fact illustrates that clinicians should avoid using diagnostic criteria as a tick-box exercise in the search for diagnosis.

By presenting this case at the Case-Based Conference we are aiming to raise awareness about MAS in the context of PsA and learn how other teams approach patients with known autoimmune conditions and fever of unknown origin.

The authors received no financial support for the research, authorship, and publication of this article.

